# Viroids and Satellites and Their Vector Interactions

**DOI:** 10.3390/v16101598

**Published:** 2024-10-11

**Authors:** Ahmed Hadidi, Henryk H. Czosnek, Kriton Kalantidis, Peter Palukaitis

**Affiliations:** 1 U.S. Department of Agriculture, Agricultural Research Service, Beltsville, MD 20705, USA; 2 Institute of Plant Sciences and Genetics in Agriculture, The Robert H. Smith Faculty of Agriculture, Food and Environment, The Hebrew University of Jerusalem, Rehovot 76100, Israel; hanokh.czosnek@mail.huji.ac.il; 3 Department of Biology, University of Crete, 70013 Heraklion, Greece; kriton@imbb.forth.gr; 4 Institute of Molecular Biology and Biotechnology, Foundation of Research and Technology-Hallas (FORTH), 70013 Heraklion, Greece; 5 Graduate School of Plant Protection and Quarantine, Jeonbuk National University, Jeonju 54896, Republic of Korea; scripath1@yahoo.co.uk

## 1. Introduction

Many diseases of unknown etiology with symptoms like those caused by plant viruses but for which no virions could be found were described during the early and mid-20th century. In 1971, T.O. Diener demonstrated that the causal agent of potato spindle disease, first described in the early 1920s, is a free RNA of 25,000–110,000 Daltons, which is much smaller than a viral genome, and that no viral coat proteins were synthesized in infected plants [1,2]. He concluded that the RNA is too small to contain the genetic information necessary for self-replication and that it must rely on host enzymes for its replication. In 1972, Joseph S. Semancik and L. G. Weathers reported similar findings for the causal agent of citrus exocortis disease [3]. For such an unconventional agent with symptomatology reminiscent of viruses, the term viroid, suggested by Diener [2], was adopted in 1972. Diener remarked that the term viroid had earlier been put forward by Altenburg in 1946 to describe possible “…symbionts akin to the viruses…” in animal cells [4]. Viroids are small, covalently closed circular or linear highly structured RNA molecules with a high degree of base pairing, replicate autonomously, do not encode proteins, and use pre-existing host-cell RNA polymerase and processing enzymes for replication and pathogenesis [5,6,7]. Viroids belong to the order of *subviral agents*, which currently includes two families, eight genera, and 43 species that have been biologically and molecularly characterized [8].

The natural host range of viroids includes vegetables, field and ornamental crops, fruit and palm trees, and grapevines [5,6,7]. Recently, it has been shown that apple scar skin viroid naturally infects several species of filamentous fungi in viroid-infected apple trees [9]. Moreover, under laboratory conditions, a few viroids replicated in filamentous fungi [10]. The term mycoviroids was coined by Ahmed Hadidi in 2022 for viroids that infect plant pathogenic fungi [11].

Viroids have had an impact on the science of virology, plant pathology, botany, microbiology, genetics, genomics, molecular biology, and molecular evolution as they represent the progenitors of life since they are infectious agents consisting of only 234–401 ribonucleotides [12].

Ted Diener and Stanley Prusiner [13] compared the properties of viroids with those of the scrapie agent that Prusiner purified and named prions [14]. Viroids are infectious RNAs that infect plants and fungi [5], but prions, proteinaceous infectious particles, are the agents of sheep scrapie disease, human kuru and Creutzfeldt–Jakob diseases, and some other spongiform encephalopathy diseases [15]. Prions are solely host-encoded proteins that cause disease by misfolding, aggregation, and transmission of their conformations into infectious prion isoforms [16]. Prusiner was awarded the Nobel Prize in Medicine “for his discovery of Prions—a new biological principle of infections” in 1997.

In 1969 and 1971, Irving R. Schneider described that an RNA dependent on a helper virus for replication and movement was found in association with tobacco ringspot virus and was named “satellite RNA” [17,18]. In 1972, a devastating epidemic disease swept through the tomato fields of the French Alsace province, destroying the crop. The causal agent was not revealed until 1977, when Jacobus M. Kaper clearly demonstrated that RNA 5 of cucumber mosaic virus (CMV) was required to cause the Alsace disease [19]. Because RNA 5 alone cannot cause the disease but needs other CMV RNAs for replication [20], this CMV RNA was recognized as the first RNA satellite to cause a plant disease. This syndrome arose again in the 1980s and 1990s in various European countries, as well as in Japan. The DNA satellites of geminiviruses were described about two decades later. The first one identified in 1997 [21] was found to be a defective form of a larger satellite designated DNA beta in 2000 [22], one of many since described and now referred to as the betasatellites of begomoviruses.

In general, with a few exceptions, most satellites are considered unique and different from either sub-genomic RNAs or viral defective interfering RNAs/DNAs with sequences identical to and derived from part(s) of the viral genome [23]. Historically, satellites were not necessary for the replication of their helper viruses and were thus considered completely redundant. This view, however, has changed to some extent after more than two decades, when satellite-like RNAs were found to be required for their helper viruses’ specific movement within the infected host plant, plant-to-plant transmission, or plant-to-vector transmission [23].

The alphasatellites of both geminiviruses and nanoviruses are small circular DNAs that depend on their associated viruses for encapsidation, movement, and transmission; however, they are capable of autonomous replication. Hence, they are not true satellites and will not be covered.

This Special Issue commemorates the work of Ted Diener and others involved in the discovery and characterization of various viroids and other sub-viral agents; viz., satellite RNAs, DNA betasatellites, and prions, with a series of articles describing the history and distribution of these pathogenic molecules, as well as a new work in these areas.

## 2. VIROIDS

### 2.1. The Remarkable Legacy of Theodor O. Diener (1921–2023): Preeminent Plant Pathologist and the Discoverer of Viroids [24]

The infectious nature of potato spindle tuber disease was demonstrated by Schultz and Folsom in the 1920s. In 1967 Theodor, (“Ted”) Otto Diener and William Raymer obtained evidence that the agent of this disease was not a virus but rather a very small, protein-free RNA. Four years later, Ted Diener conclusively demonstrated in two landmark articles that the causal agent was an infectious, autonomously replicating, and very small single-stranded RNA, which he termed “viroid” to differentiate it from viruses.

The discovery of viroids in 1971 represents the third major extension in the history of the biosphere, following the earlier discoveries of “subvisible” microorganisms by the Dutch microbiologist Antonie van Leeuwenhoek in 1675 and of “submicroscopic” viruses by the Russian botanist Dmitri I. Ivanovsky in 1892. The discovery of the potato spindle tuber viroid (PSTVd) also revealed the existence of previously unsuspected regulatory pathways in plants. Over the succeeding 50+ years, 42 additional species of viroids have been isolated from a wide variety of vegetable and ornamental crops, including fruit trees, palms, and grapevine. The International Committee on Virus Taxonomy (ICTV) recognized the discovery of viroids by establishing a new order of subviral agents that includes two families: *Pospiviroidae* with five genera, and *Avsunviroidae* with three genera.

In his 2003 article “Discovering viroids-a personal perspective”, Ted Diener describes how he combined then state-of-the-art technology (i.e., ultracentrifugation and polyacrylamide gel electrophoresis) with tomato bioassays to demonstrate that the causative agent of this disease could not be (i) a plant virus whose coat protein is either non-functional or yields only very unstable virions, (ii) a satellite RNA whose replication is totally dependent on the presence of a helper virus, or (iii) a collection of small RNAs that together could represent a viral genome of conventional size.

Similar agents were isolated from two other crop species, i.e., citrus exocortis viroid (CEV) and chrysanthemum stunt viroid (CSV), and just seven years later, in Germany, Heinz Sänger and colleagues published the complete nucleotide sequence of PSTVd. Notably, PSTVd was the first eukaryotic pathogen to have its genome completely sequenced.

The fact that a low molecular weight RNA could replicate autonomously in a variety of host species apparently uninfected by another agent had a number of important implications for virology, molecular biology, and genetics. The molecular processes responsible for viroid replication, movement, and pathogenicity have been thoroughly investigated in many laboratories worldwide. It was concluded that viroids are small, covalently closed, circular or linear highly structured RNA molecules with a high degree of base pairing, replicate autonomously, do not encode proteins, and use pre-existing host-cell RNA polymerase and processing enzymes for replication and pathogenesis.

The accelerating pace of molecular studies involving viroids produced an ever-increasing number of complete viroid nucleotide sequences, thereby allowing Ted to turn his attention to the question of viroid origin. In 1989, he proposed that viroids and certain viroid-like satellite RNAs may be “living fossils” from a pre-cellular RNA world. Consistent with such a scenario, initial phylogenetic analyses indicated a monophyletic origin for viroids, selected plant satellite RNAs, and the viroid-like domain of human hepatitis delta virus (HDV) RNA. The small size and circularity of viroids would have enhanced the probability of survival under the primitive error-prone conditions faced by self-replicating RNA systems and assured complete replication without the need for initiation or termination signals.

Although leaving the apparent absence of viroids from prokaryotic algae (ancestors of modern higher plants) unexplained, this RNA world hypothesis is still widely accepted. Recently, however, the outlines of an alternative hypothesis began to emerge. Two independent metagenomic analyses of environmental samples indicate that fungi provide an evolutionary hub for RNA viruses and viroid-like elements. Furthermore, evidence has been presented for the transmission of the apple scar skin viroid from plants to plant-associated fungi under natural conditions.

Ted Diener’s many contributions to biological science have been widely recognized by the American Phytopathological Society, the Alexander von Humboldt Foundation, the U.S. National Academy of Sciences, the American Academy of Arts and Sciences in the 1970s, and the Leopoldina (German Academy of Sciences) in 1980. In 1987, he was awarded the E.C. Stakman Award (University of Minnesota), the Wolf Prize in Agriculture (Israel), and the National Medal of Science (USA). He was inducted into the Agricultural Research Service Science Hall of Fame in 1989. In 2000, the American Phytopathological Society declared his discovery of the viroid to be one of the six most important discoveries of the millennium to involve pathogens.

### 2.2. Viroids of the Mediterranean Basin [25]

The Mediterranean basin (also known as the Mediterranean Region) is the region of land around the Mediterranean Sea with a coastline of about 46,000 km (about 28,600 miles). The Mediterranean basin covers portions of three continents, north Africa, southwest Asia, and south Europe, that include 22 countries with a population of about 500 million people. These countries include Albania, Algeria, Bosnia and Herzegovina, Croatia, Cyprus, Egypt, France, Greece, Israel, Italy, Lebanon, Libya, Malta, Monaco, Montenegro, Morocco, the Palestinian Authority, Slovenia, Spain, Syria, Tunisia, and Turkey.

Twenty-nine viroids were identified and characterized in the Mediterranean basin. Viroids that have been reported in woody plants such as fruit trees (pome, stone, citrus, fig, mulberry, avocado, mango, pomegranate), nut trees (walnut, almond, pistachio), and grapevine belong to six viroid genera: *Pelamoviroid*: apple hammerhead viroid (AHVd), peach latent mosaic viroid (PLMVd); *Avsunviroid*: avocado sunblotch viroid (ASBVd); *Hostuviroid*: hop stunt viroid (HSVd); *Apscaviroid*: apple dimple fruit viroid (ADFVd), apple scar skin viroid (ASSVd), Australian grapevine viroid (AGVd), citrus bent leaf viroid (CBLVd), citrus dwarfing viroid (CDVd), citrus viroid V (CVd-V), grapevine latent viroid (GLVd), grapevine yellow speckle viroid 1 (GYSVd-1), grapevine yellow speckle viroid 2 (GYSVd-2), pear blister canker viroid (PBCVd); *Cocadviroid*: citrus bark cracking viroid (CBCVd); and *Pospiviroid*: CEVd.

ASSVd and ADFVd are prevalent in pome fruits, whereas HSVd and PLMVd are prevalent in stone fruits. PLMVd has been found frequently in peach and occasionally in plum, sweet cherry, and apricot germplasm in very significant percentages. HSVd has also been frequently detected in several stone fruit species, like apricot, peach, plum, nuts like almond, other fruit trees like fig, pomegranate, and mulberry, as well as in grapevine. HSVd is the viroid with the widest host range. Despite its infection being usually latent in stone fruits, it has been associated with serious disorders of economic importance; the first is the dapple fruit disease of plum and peach, “kirin-ka”, and the second, an apricot fruit disorder known as “fruit degeneration”, characterized by fruit rugosity and the loss of organoleptic properties.

In grapevines, although viroids usually do not cause severe symptoms, some of them are disease agents in certain environmental conditions or in combination with certain viruses, and some other viroids, like HSVd or CEVd, may cause severe diseases in other crops.

In citrus fruit trees, CEVd and HSVd have been reported in most Mediterranean countries and are among the most prevalent citrus viroids in the region because the usual citrus rootstock (sour orange) is tolerant to diseases caused by them, namely, citrus cachexia (xyloporosis) and citrus gummy bark, which are caused by variants of HSVd, and citrus exocortis, caused by the eponymous *Pospiviroid*. These diseases pose a threat to the citrus tristeza virus (CTV)-tolerant trifoliate rootstocks used, as these plants are susceptible to those viroids.

Viroids infecting herbaceous and ornamental crops in the Mediterranean basin belong to six viroid genera: *Pelamoviroid*: chrysanthemum chlorotic mottle viriod (CChMVd), PLMVd; *Elaviroid*: eggplant latent viroid (ELVd); *Hostuviroid*: HSVd; *Cocadviroid*: hop latent viroid (HLVd); *Coleviroid*: coleus blumei viroid- 1 (CbVd-1); coleus blumei viroid- 3 (CbVd-3); and *Pospiviroid*: PSTVd, CEVd, CSVd, columnea latent viroid (CLVd), iresine viroid 1 (IrVd-1), tomato apical stunt viroid (TASVd), and tomato chlorotic dwarf viroid (TCDVd). These viroids, with the exception of HLVd, have been reported in vegetables (tomato, potato, pepper, eggplant, carrot, broad bean, chickpea, cucumber), oil crops (rapeseed), several ornamental crops like chrysanthemum, marguerite daisy, common ragwort, cape gooseberry), spur flower, common periwinkle, petunia, trailing petunia, and climbing nightshade, as well as in sorghum, the latter being the first report of a viroid infecting poaceous monocots in the Mediterranean and worldwide. Hops are infected by HLVd (causes hop latent), CBCVd (causes severe hop stunt), and HSVd (causes hop stunt). PSTVd has been detected in several ornamental cape gooseberry, common ragwort, petunia, night blooming jessamine, trailing petunia, climbing nightshade, angel trumpets, herbaceous crop seeds (tomato, pepper, eggplant), and tubers of potato in some Mediterranean countries and in the rest of Europe.

Not only have detection methods, such as reverse transcription-quantitative polymerase chain reaction and next-generation sequencing, been used for viroid detection, along with molecular hybridization techniques allowing for rapid detection, identification, and characterization of known and novel viroids in these countries, but also eradication measures have been taken that allowed for the efficient elimination of certain viroids in a number of Mediterranean countries. The eradication measures were followed as recommended by the European and Mediterranean Plant Protection Organization, which is known by its abbreviation, EPPO. The Mediterranean Region has been a niche for viroids since ancient times due to the warm climate and the socio-cultural conditions that facilitate viroid transmission among different host plant species.

Other known viroids have not been identified in the Mediterranean basin. They include apple chlorotic fruit spot viroid, apple fruit crinkle viroid, citrus viroid VI (CVd-VI), coconut cadang-cadang viroid, coconut tinangaja viroid, coleus blumei viroid-5, coleus blumei viroid-6, coleus blumei viroid-7, pepper chat fruit viroid, portulaca latent viroid, persimmon viroid-2, and tomato planta macho viroid.

### 2.3. New Insights into Hop Latent Viroid Detection, Infectivity, Host Range, and Transmission [26]

HLVd which infects commercial hops (*Humulus lupulus* L.), was discovered in the late 1980s in Spain. Hops are propagated vegetatively, and the efficient mechanical transmission of HLVd, in combination with the absence of acute symptoms, has contributed to its worldwide distribution.

Recently, growers have reported devastating economic losses due to “dudding disease” caused by HLVd in industrial hemp (*Cannabis sativa* L.). In mature hop and hemp cones, the secondary metabolite contents are greatly affected, and the cone yield is significantly reduced. Losses to the hemp industry were estimated to be four billion dollars per year.

Because of its latency, HLVd has no apparent symptoms in young hemp and hop plants and can go undetected. Industrial hemp plants are often clonally propagated, and there is a need for new methods to assess their phytosanitary status before vegetative propagation.

The current study reported sensitive RT-PCR detection of HLVd using primers designed to detect HLVd RNA for simple interpretation of PCR products by agarose gel electrophoresis. As little as 5 pg of total RNA were used for the viroid detection. A survey of hemp samples obtained from a diseased production system proved sole infection of HLVd (72%) with no coexistence of HSVd. HLVd was infectious through successive passage assays using a crude sap or total RNA extract derived from infected hemp. HLVd was also highly transmissible through hemp seeds at rates of 58 to 80%. Moreover, HLVd-containing sap or RNA preparations from HLVd-infected hemp plants inoculated to plants belonging to six species revealed a wider host range of plant species than previously reported. The study also confirmed vertical transmission of HLVd through seeds as a significant means of disease dispersal.

In this study, new hosts for HLVd were reported, which include tomato, cucumber, chrysanthemum, *Nicotiana benthamiana*, and *Arabidopsis thaliana* (Col-0). In addition, sequence analysis of 77 HLVd isolates revealed only three parsimony-informative sites, while 10 sites were detected among all HLVd isolates available in the GenBank. The phylogenetic relationship among HLVd isolates allowed for inferring two major clades based on the genetic distance. These findings facilitate further studies on host–viroid interaction and viroid management.

## 3. Understanding Citrus Viroid Interactions: Experience and Prospects [27]

Citrus trees are natural hosts of at least eight viroids, including CEVd, CBLVd, HSVd, CDVd, CBCVd, CVd-V, CVd-VI, and citrus viroid VII (CVd-VII). These viroids are widely present and often infect citrus orchards around the world in combination and exhibit complex interactions during mixed infection. There are two opposite types of interactions among citrus viroids co-infecting the same plant, leading to antagonistic or synergistic effects.

The antagonistic effect, which is also known as cross-protection, leads to reduced viroid symptoms due to a decrease in viroid pathogenicity, while the synergistic effect leads to an increase in symptom severity because of an increase in viroid pathogenicity. Understanding and analyzing the molecular mechanisms of viroid pathogenicity induced during these interactions are of great significance for preventing and controlling citrus viroid diseases. This study points out the core role of the host RNA silencing mechanism and viroid-derived small interferingRNA (vd-siRNA).

The antagonistic effect among viroids refers to the ability of viroids to infect the host being influenced by the pre-infection of other strains of the same or closely related viroids. Specifically, when citrus plants pre-infected with a mild CEVd strain are inoculated with a severe strain of CEVd, the typical symptoms of the severe strain and its RNA accumulation level will be blocked or weakened for a period. The level of protection depends on the inoculation interval between mild and severe strains. It was also found that trees infected with a mixture of CEVd and CBCVd showed an antagonistic effect in terms of bark scaling and cracking symptoms. CEVd can cause severe tree dwarfing and bark cracking of trifoliate orange rootstocks, both of which are characteristics of exocortis disease. When the viroid mixture contains CBCVd, the symptoms of bark cracking are alleviated or even suppressed. The impact of CBCVd on citrus growth and fruit yield is minimal. Therefore, CBCVd can serve as an antagonistic factor for the symptoms of CEVd, and CBCVd can reduce or inhibit the effects of CEVd on citrus growth and yield. CBCVd is infectious to both citrus and hops, and its genome has sequence homology with HSVd in the left terminal region, while it has high sequence homology with CEVd in the right terminal region. Therefore, CBCVd is considered a chimeric recombinant of CEVd and HSVd.

Previous studies have shown that viroid RNA is an activator and target of RNA silencing in hosts. The subsequently generated vd-siRNA also plays a crucial role in the pathogenesis of the viroid by targeting the RNA transcripts of host genes. Gene silencing largely explains why cross-protection only occurs among viroids with sequence homology. There are reasons to believe that the cross-protection of viroids depends on the sequence homology of vd-siRNAs entering the RNA-induced silencing complex (RISC). Due to the common sequence and structural characteristics between the CEVd and CBCVd genomes, the antagonistic effect of CBCVd on CEVd may be related to RNA silencing and vd-siRNA.

The identification of small RNAs of citrus viroids is of great value for understanding the interaction mechanism among citrus viroids. It was demonstrated that CBCVd attenuates the symptoms and accumulation of CEVd through the host’s RNA silencing mechanism. Based on current experience, CBCVd and CEVd can produce many identical vd-siRNAs in the homologous region when infecting citrus. When CBCVd and CEVd co-infect citrus, the homologous region is the hotspot region for vd-siRNA production. It is worth noting that there is an antagonistic effect between CBCVd and CEVd in citrus, while there is a synergistic effect between CBCVd and HSVd. CBCVd is homologous with HSVd in the left terminal region and highly homologous with CEVd in the right terminal region. These phenomena indicate that the interaction among citrus viroids is very complex. The performance of the final interaction results may be influenced by multiple factors such as the viroid sequence, viroid structure, and host species.

### 3.1. The Secondary Structure of Potato Spindle Tuber Viroid Determines Its Infectivity in Nicotiana benthamiana [28]

Potato spindle tuber viroid (PSTVd) was used as a model to achieve an understanding of the relationship between RNA secondary structure and function. PSTVd consists of 359 nucleotides and adopts a rod-like structure characterized by 27 loops and 26 stems or helices. Specific loop and stem structures have been identified as key factors influencing the directional movement of PSTVd among various cell types. Through the generation of linear RNA transcripts with distinct start sites (representing different PSTVd forms), a range of PSTVd secondary structures were created while retaining the identical sequence. The RNA secondary structures were predicted using the mfold tool and validated through native polyacrylamide gel electrophoresis after in vitro RNA folding. Analyses revealed that the majority of the secondary structures exhibited a rod-like conformation. However, PSTVd forms beginning at positions 17, 250, and 265 displayed a distinctive ‘+’ or ‘Y’ shape. Plant infection assays using *Nicotiana benthamiana* plants, with six plants assigned to each form. The inoculum was carried out through rub-inoculation, 300 ng PSTVd in vitro transcripts per plant, and systemic infection was checked four weeks later. Results revealed that the formation of a proper rod-like structure is crucial for the successful infection of PSTVd. The inability of PSTVd forms with non-rod-like structures to infect plants could be partially compensated by increasing the amount of linear viroid RNA transcripts, suggesting the existence of additional RNA secondary structures, such as the correct rod-like structure, alongside the dominant structure in the RNA inoculum of these forms. This study demonstrates the critical role of PSTVd secondary structures in determining the function of its infectivity.

### 3.2. Small Heat Shock Protein (sHsp 22.98) from Trialeurodes vaporariorum Plays an Important Role in Apple Scar Skin Viroid Transmission [29]

The greenhouse whitefly *Trialeurodes vaporariorum* infests many crops and is a vector for transmission of ASSVd. The whitefly’s proteins, which interact with the viroid, were investigated for effective management of ASSVd. It was revealed that the whitefly synthesizes, under stress, a small heat shock protein (sHsp), which binds to ASSVd. The *sHsp* gene is 606 bp and encodes for 202 amino acids, with a molecular weight of 22.98 kDa. The sHsp22.98 protein was found to exist in both monomeric and dimeric forms, and both forms showed strong binding to ASSVd. To investigate the role of sHsp22.98 during ASSVd infection, transient silencing of *sHsp22.98* was conducted, using a tobacco rattle virus induced gene silencing system. The *sHsp22.98*-silenced whiteflies showed an approximate 50% decrease in ASSVd transmission. These results suggest that sHsp22.98 from *T. vaporariorum* is associated with ASSVd and plays a significant role in its transmission.

## 4. Development of a CRISPR/SHERLOCK-Based Method for Rapid and Sensitive Detection of Selected Pospiviroids [30]

The potential application of the CRISPR-Cas13a system for the rapid and accurate diagnostic assay for known viroids was suggested by Hadidi in January 2019 in an article published in Viruses [31]. Cas13 is an RNA-guided RNase that cleaves single-stranded and collateral RNAs. The CRISPR/Cas system will work only for known viroids in contrast to next-generation sequencing (NGS), but it should be much faster. Currently, viroids are detected by NGS and real-time RT-PCR.

A method was developed for the rapid Cas13a-based Specific High-sensitivity Enzymatic Reporter unLOCKing (SHERLOCK) platform for detection of pospiviroids. The limits of detection and specificity of CRISPR-Cas13a assays were determined. This platform combines recombinase polymerase amplification (RPA) with CRISPR and CRISPR-associated (CRISPR-Cas) RNA-guided endoribonuclease that is rapid and does not require expensive equipment; it is highly specific in differentiating closely related pospiviroids; and it can be adapted for on-site detection.

The developed SHERLOCK platform was used to detect six pospiviroids of quarantine importance in the United States. The viroids include PSTVd, TASVd, TCDVd, tomato planta matcho viroid, pepper chat fruit viroid, and CLVd. This is the first report of using SHERLOCK to detect pospiviroids of quarantine interest.

## 5. VIROIDS, SATELLITE RNAs, and PRIONS

### Viroids, Satellite RNAs, and Prions: Folding of Nucleic Acids and Misfolding of Proteins [32]

The history of viroid discovery as well as its properties by Ted Diener in 1971 and 1972 have already been detailed. In the 1970s and 1980s, viroids were often discussed at conferences together with other “subviral pathogens”. This term includes what are now called satellite RNAs and prions. Satellite RNAs depend on a helper virus and have linear or, in the case of virusoids, circular RNA genomes. Prions, proteinaceous infectious particles, are the agents of sheep scrapie disease, human kuru disease, and other spongiform encephalopathy diseases. Many satellite RNAs, like viroids, are non-coding and exert their function by thermodynamically or kinetically controlled folding, while prions are solely host-encoded proteins that cause disease by misfolding, aggregation, and transmission of their conformations into infectious prion isoforms. The pioneering work of Ted Diener on viroids has been presented in detail in an article by Owens and Hadidi in this editorial.

Satellite RNAs were discovered at about the same time as viroids. In 1971, Irving Schneider gave the name “satellite RNA” to the satellite RNA of tobacco ringspot virus (TRSV), which depends on TRSV for its replication and source of coat protein. The satellite reduces TRSV accumulation and the severity of the virus-induced symptoms. In 1977, Kaper and Waterworth demonstrated that CMV-associated RNA 5 was the causal agent for tomato necrosis disease, which caused a devastating disease epidemic that swept through tomato fields of the French Alsace province, destroying the crop for five years. Since CMV RNA 5 alone cannot cause the disease but needed all three genomic RNAs of CMV for replication, CMV RNA 5 was recognized as the first RNA satellite to cause a plant disease. The term virusoid has been used to describe small, circular, single-stranded satellite RNAs that are encapsidated by respective plant viruses and replicated by the viral polymerase; their native secondary structure is mostly rod-like with a few small bifurcations RNAs. These circular satellite RNAs, all associated with viruses that are members of the genus *Sobemovirus*, have viroid-like structures and contain ribozymes involved in the self-cleavage of concatemeric RNAs produced during their rolling circle replication. In contrast to viroids, virusoids are thermodynamically less stable, despite similar GC content, and do not possess extra-stable hairpin(s).

The proteinaceous particles, called prions, were first discovered in studies of the infectious agent of scrapie disease in sheep. In 1978, Stanley B. Prusiner reported that the sedimentation behavior of scrapie infectivity was heterogeneous and different from viruses and viroids. Prusiner summarized the results of his subsequent studies: chemical and physical procedures that destroy nucleic acids do not destroy scrapie infectivity, while chemical and physical procedures that destroy proteins do destroy scrapie infectivity. In his landmark publication in 1982, Prusiner concluded, “Novel proteinaceous infectious particles cause scrapie”. Prusiner was awarded the Nobel Prize in Physiology or Medicine in 1997. Prize motivation: “for his discovery of Prions-a new biological principle of infection”. Seven years later, in 2004, a synthetic prion—i.e., a synthetic protein that never has seen an animal—was created.

The prion protein (PrP) can exist in a cellular, non-pathological conformation (PrP^c^), which is mainly *α*-helical, or in an aggregated, pathological conformation (PrP^Sc^, i.e., scrapie). The infectivity of prions results from a conformational change in prion protein PrP^c^ into PrP^Sc^.

In the early 1980s, there was little interest in rare neurodegenerative diseases. This situation changed significantly with Prusiner’s concept of prions and the discovery of the bovine spongiform encephalopathy (BSE) epidemic in the mid-1980s. A few years later, human Creutzfeldt–Jakob disease was found to be caused by BSE prions. With widespread testing of slaughtered cattle in Europe and elimination of bovine offal as a source of feed for cattle, sheep, and pigs, BSE was eliminated from the roster of lethal human illnesses.

Our increased knowledge of prions has led to effective therapeutics for Alzheimer’s and related human diseases. State-of-the-art technologies are helping to elucidate the fibril structures of proteins that cause prion diseases. By understanding the biophysics, molecular biology, and protein interactions involved in these diseases, effective therapeutics can be developed.

What started with research in plants and animals had a later impact in general molecular biology, extending to human disease and therapeutic developments. Sidney Altman and Thomas R. Cech discovered ribozymes in the 1980s, and George Bruening discovered the hammerhead ribozyme in 1990 while studying the satellite RNA of the tobacco ringspot virus. The hammerhead ribozyme is a self-cleaving, non-coding RNA that can cleave target mRNA sequences and produce products that are degraded in the cell. The hammerhead ribozyme is characterized by a two-dimensional structural motif called the “hammerhead” that allows for site-specific cleavage. Robert H. Symons was the first to describe self-cleavage of avocado sunblotch viroid (ASBVd) in 1986. Members of the viroid family *Avsunviroidae* (type member ASBVd) have hammerhead ribozymes in both the genomic and antigenomic RNAs. Hammerhead ribozymes have been detected in most genomes. Retrotransposons with hammerhead ribozymes, called retrozymes, have been found encoded in diverse plant genomes and have stimulated new ideas about the possible origin of viroid and viroid-like RNAs. Viroids, termed mycoviroids, have been detected in fungi. Electron microscopy examination of viroids in the 1970s revealed that viroids are covalently closed circular RNA molecules. Thus, viroids were the first discovered circular RNAs. Knowledge of circular RNAs in mammals has expanded in recent years; for example, they are produced by a process called back-splicing from mRNAs and are involved in the (mis)regulation of many processes. Current knowledge on virusoids and other satellite RNAs has expanded substantially.

Numerous autonomous non-hepatitis B virus-dependent HDV satellites have been identified in other mammals recently, suggesting a common origin not only for viroids and viroid-like satellite RNAs but also for HDV.

## 6. The Role of Satellites in the Evolution of Begomoviruses [33]

Begomoviruses have emerged prominently during the last four decades as the most successful group of devastating plant viruses, affecting a wide range of dicotyledonous crops worldwide, particularly in the tropics and subtropics, causing enormous economic losses and threatening food security. Epidemics caused by begomoviruses have spread in regions and crops that were previously free from these viruses. The most seriously affected crops include cassava; cotton; grain legumes; and cucurbitaceous, malvaceous, and solanaceous vegetables. Despite efforts to contain begomoviruses, they continue to emerge and re-emerge in diverse crops and regions.

Three types of circular ssDNA satellite molecules, termed alphasatellites, betasatellites, and deltasatellites, are associated with the diseases caused by begomoviruses, but begomovirus–betasatellite complexes have played significant roles in the evolution of begomoviruses, causing widespread epidemics in many economically important crops throughout the world. Betasatellites play a significant role in increasing the virulence of begomoviruses, whereas alphasatellites and deltasatellites do not seem to play any noticeable role in viral pathogenesis.

Begomoviruses and their satellites are efficiently transmitted by the polyphagous whitefly (*Bemisia tabaci*). The genome of begomoviruses is either monopartite or bipartite and consists of circular ssDNA molecules encapsidated in twinned (geminate) particles. The begomoviruses spreading in the New World are mostly bipartite, whereas those spreading in the Old World have both bipartite and monopartite genomes.

Begomoviruses seem to have acquired DNA satellites to gain an evolutionary advantage, as most betasatellites enhance the accumulation of viral DNA. The satellites, in general, may have originated from genetic elements that were once part of the viral genome but eventually lost their ability to replicate independently. Over time, these genetic elements may have become dependent on helper viruses to provide the necessary machinery for their replication and transmission. The evolutionary origin of betasatellites is not clearly understood, although it was suggested that these may be genetic remnants of some unidentified or extinct viruses. However, the greater molecular diversity of betasatellites occurring in the Indian subcontinent and China indicates that this region may be the center of origin of the betasatellites of begomoviruses.

Other factors, like genetic mutations, recombination events, and selection pressures, may also have driven the evolution of viral satellites by enhancing their ability to interact with the helper viruses and evade host defenses. Recombination events between different satellite and helper virus genomes can also contribute to the generation of new satellite variants. Selection pressures also play a crucial role in the evolution of viral satellites. Begomovirus satellites seem to have efficiently exploited the resources of helper viruses to persist, spread, and disarm the host resistance by evading detection and/or manipulation of the host immune system.

Begomovirus-associated satellites, particularly betasatellites, have played a significant role in countering plant defense. The multifunctional βC1 protein encoded by betasatellites interacts with cellular proteins and modulates several cellular processes, especially targeting the innate immune system of plants. The βC1 protein was shown to suppress plant defense mechanisms, such as post-transcriptional gene silencing, hormone-based defense systems, ubiquitin-mediated proteome degradation system, and autophagy. Betasatellites played a significant role in the evolution of begomoviruses by their ability to increase disease severity by disarming host resistance and improving transmissibility of the viruses by the vector whitefly.

Mixed infections by monopartite and bipartite begomoviruses in the Indian subcontinent may have helped the development of the genetic diversity of begomoviruses, rapid adaptation of the viruses and their satellites to new environments, and expanding the virus–host range. The novel DNA satellites associated with bipartite begomoviruses in the New World were found to share some genetic features with betasatellites and contain nucleotide stretches of begomoviral origin, presumably the remains of recombination events involved in their origin.

The global spread of betasatellites shows China, India, and Pakistan to be hotspots for betasatellites, with limited occurrence of betasatellites in other parts of the Old World. The hotspots for betasatellites in Asia may have developed due to the intensive cropping systems and more favorable environmental conditions, ideal for the perpetuation of begomoviruses and their vector *B. tabaci*. The New World is nearly free from betasatellites.

Understanding of the processes by which begomoviruses and their satellites disarm the host defense will be useful in developing strategies for the management of diseases caused by begomoviruses. The challenge is to develop effective strategies to minimize the damage caused by begomovirus complexes in various cropping systems around the world.

### 6.1. Identification of Host Factors Interacting with a γ-Shaped RNA Element from a Plant Virus-Associated Satellite RNA [34]

Some plant viruses have been found to be associated with satellite RNAs (satRNAs), including CMV, turnip crinkle virus, and others. One of the well-studied satRNAs is the CMV-associated satRNA, which is the second satRNA reported. CMV is an economically important plant virus, infecting over 1200 plant species. CMV has a tripartite single-stranded RNA genome (RNAs 1–3) that encodes five viral proteins. A number of CMV isolates or strains are associated with satRNAs that vary in size, ranging from 330 to 405 nucleotides. Infections involving satRNAs cause profound alterations to CMV-induced disease symptoms in host plants. In a few cases, viral diseases are intensified in the presence of a satRNA isolate. However, the majority of CMV satRNAs reduced viral titers in host plants, leading to the amelioration of viral symptoms. This symptom amelioration is proposed to be the consequence of either the competition of satRNA with CMV RNAs for the viral replicase or the interference with the suppressor activity or the decreased expression level of the CMV 2b protein.

Previously, a highly conserved RNA element with a γ-shaped structure (γSS) in CMV satRNAs was identified, and this element was referred to as a γ-shaped RNA element (γRE). γRE is indispensable for satRNA survival and required for satRNA to inhibit the replication of CMV RNA1 and RNA2. γRE was proposed to competitively bind host factors that are required for the replication of viral genomic RNAs. In the present work, pull-down assays were used to screen candidates of host factors from *Nicotiana benthamiana* plants using biotin-labeled γRE as bait. Nine host factors were found to interact specifically with γRE. Subsequently, these host factors were down-regulated individually in *N. benthamiana* plants via tobacco rattle virus-induced gene silencing and tested with infection by GFP-expressing CMV (CMV-gfp) and the isolate T1 of satRNA (sat-T1). Out of nine candidates, three host factors, namely histone 3 (H3), small GTPase Ran3 (Ran3), and eukaryotic initiation factor 4A (eIF4A), were extremely important for infection by gfp-expressing CMV and satRNA. Moreover, it was found that cytosolic glyceraldehyde-3-phosphate dehydrogenase 2 contributed to the replication of CMV and satRNA, but also negatively regulated CMV 2b activity. Collectively, the present work provides essential clues for uncovering the mechanism by which satRNAs reduce or inhibit CMV replication.

### 6.2. Molecular Signature of a Novel Alternanthera Yellow Vein Virus Variant Infecting the Ageratumconyzoides Weed in Oman [35]

It has been known that alternanthera yellow vein virus (AlYVV), a monopartite *begomovirus*, infects a diverse range of crops and native plants in South Asia. The virus was initially discovered in China in 2005, primarily infecting *Alternanthera philoxeroides.* The virus was then reported from Vietnam in 2008, Pakistan in 2010, and India in 2019. Recently, distinctive yellow vein symptoms, characteristic of *begomovirus* infection, have been observed on the weed *Ageratum conyzoides* in Oman, which prompted a thorough genomic characterization in this study. The results revealed a complete genome sequence of 2745 base pairs and an associated betasatellite spanning 1345 base pairs. In addition, Sequence Demarcation Tool analyses indicated the highest nucleotide identity of 92.5–92.8% with Indian virus isolates, whereas the betasatellite exhibited a 99.8% nucleotide identity with isolates of the tomato leaf curl betasatellite. The association of betasatellite may enhance AlYVV ability to overcome host defense responses, broaden host ranges, and indirectly impact *in planta* virus accumulation. The very close genetic relationship between AlYVV Oman virus (AlYVV-OM) and Indian isolates suggests a likely origin from India, with the possibility that trade via sea or air routes facilitated the distribution of this virus into Oman. These findings propose a novel AlYVV-OM variant and emphasize the need for additional epidemiological surveillance to understand its prevalence and significance in Oman and the broader region and to implement phytosanitary protective measures to curtail its transmission.
